# High Density Lipoproteins and Diabetes

**DOI:** 10.3390/cells10040850

**Published:** 2021-04-09

**Authors:** Blake J. Cochran, Kwok-Leung Ong, Bikash Manandhar, Kerry-Anne Rye

**Affiliations:** Lipid Research Group, School of Medical Sciences, Faculty of Medicine, University of New South Wales Sydney, Sydney, NSW 2052, Australia; b.cochran@unsw.edu.au (B.J.C.); kwokleung.ong@unsw.edu.au (K.-L.O.); b.manandhar@student.unsw.edu (B.M.)

**Keywords:** HDL, apoA-I, diabetes, β-cells, skeletal muscle

## Abstract

Epidemiological studies have established that a high plasma high density lipoprotein cholesterol (HDL-C) level is associated with reduced cardiovascular risk. However, recent randomised clinical trials of interventions that increase HDL-C levels have failed to establish a causal basis for this relationship. This has led to a shift in HDL research efforts towards developing strategies that improve the cardioprotective functions of HDLs, rather than simply increasing HDL-C levels. These efforts are also leading to the discovery of novel HDL functions that are unrelated to cardiovascular disease. One of the most recently identified functions of HDLs is their potent antidiabetic properties. The antidiabetic functions of HDLs, and recent key advances in this area are the subject of this review. Given that all forms of diabetes are increasing at an alarming rate globally, there is a clear unmet need to identify and develop new approaches that will complement existing therapies and reduce disease progression as well as reverse established disease. Exploration of a potential role for HDLs and their constituent lipids and apolipoproteins in this area is clearly warranted. This review highlights focus areas that have yet to be investigated and potential strategies for exploiting the antidiabetic functions of HDLs.

## 1. Introduction

Epidemiological studies have established unequivocally that a high plasma HDL cholesterol (HDL-C) level is associated with a reduced risk of having a cardiovascular event [1,2]. The strength of this relationship led to the “HDL hypothesis” which posits that the inverse association of HDL-C levels with cardiovascular risk is potentially causal, such that increasing HDL-C levels will reduce cardiovascular events [3]. The HDL hypothesis was initially supported by preclinical studies in which increasing HDL-C levels chronically in rabbits using a cholesteryl ester transfer protein (CETP) inhibitor [4] and in mice using niacin [5], or acutely by infusing synthetic, reconstituted HDL (rHDL) preparations reduced atherosclerosis [6].

However, the promise of these preclinical studies was dispelled when the “HDL hypothesis” was tested in large, randomised clinical trials of niacin and CETP inhibitors in which, with one exception, cardiovascular events were not decreased in the face of significantly increased HDL-C levels [7,8,9,10,11,12,13]. Of all of the randomised clinical trials of HDL-raising agents reported so far, the CETP inhibitor anacetrapib is the only one that that has significantly reduced cardiovascular events [14]. However, the positive outcome in that trial was largely due to a reduction in low density lipoprotein cholesterol (LDL-C) levels, not the increase in HDL-C levels. Dalcetrapib, a CETP inhibitor that modestly increases HDL-C levels and does not lower LDL-C levels [13], was found to potentially improve cardiovascular outcomes in patients carrying the AA polymorphism of the adenylate cyclase type 9 (*ADCY9*) gene [15], although this benefit could not be replicated in patients that were treated with the CETP inhibitor, evacetrapib [16]. Nevertheless, use of dalcetrapib is currently under investigation in acute coronary syndrome patients with the AA *ADCY9* genotype in a placebo-controlled, randomised, double-blind, parallel group, multicentre Phase III study [17].

Additional evidence negating the “HDL hypothesis” has emerged from Mendelian Randomisation studies, where genetically driven variations in HDL-C levels were found not to be associated with reduced cardiovascular risk [18]. However, the results of such studies should be interpreted with caution given that Mendelian Randomisation reports on linear relationships, whereas the association of HDL-C levels with cardiovascular risk is U- or J-shaped, with very low and very high HDL-C levels reported to be associated with increased mortality [19,20].

These disappointing outcomes have led to a paradigm shift in the focus of the HDL research community towards identifying other functions of HDLs and the possibility of targeting the treatment of disorders that are unrelated to cardiovascular disease. This has led to extensive investigation into a potential role for HDLs in the treatment of inflammatory diseases and diseases where oxidative stress is a key component, such as arthritis [21], cancer [20] and inflammatory bowel disease [22]. One of the most unexpected and important beneficial functions of HDLs to have emerged in recent years is the discovery that HDLs and some of their constituent apolipoproteins have potent antidiabetic properties [22,23,24,25,26,27,28].

Diabetes presents in two main forms in humans. Type 1 diabetes (T1D)**,** an autoimmune disorder in which insulin producing β-cells in the pancreas are selectively destroyed by autoreactive, proinflammatory T-cells. T1D affects approximately 10% of all patients with the disease [29,30]. T2D, by contrast, is the predominant form of the disease and affects ~90% of all patients [31,32]. T2D is driven by insulin resistance, which decreases the uptake of glucose from blood into peripheral tissues. β-cells compensate for insulin resistance and maintain blood glucose homeostasis by increasing insulin secretion [33,34]. However, prolonged β-cell compensation eventually causes β-cell death, and results in subjects with prediabetes that have impaired fasting glucose and/or impaired glucose tolerance transitioning to complete loss of β-cell function, persistently high blood glucose levels and full blown T2D [35].

In vitro and preclinical studies in animal models of diabetes, together with mounting epidemiological evidence, have generated considerable interest in the development of HDL-targeted therapies as an innovative treatment option for both T1D and T2D. As a diagnosis of diabetes is also associated with increased cardiovascular risk, it is noteworthy that therapies that increase HDL levels and additionally decrease diabetes progression and/or reverse established disease will have the added benefit of reducing cardiovascular risk.

## 2. Epidemiology of the Antidiabetic Functions of HDLs

### 2.1. Overview

A low HDL-C level has been established as a robust risk factor of diabetes in several epidemiological studies [36,37]. It has been estimated that T2D risk over a 7-year follow up period is about 4% lower per 1 mg/dL increase in HDL-C in the Framingham Offspring Study [36]. In a prospective study of 6820 nondiabetic participants from the Prevention of Renal and Vascular End-Stage Disease (PREVEND) cohort, a higher HDL-C level, a higher HDL-C/apolipoprotein A-I (apoA-I) ratio and a higher HDL-C/apolipoprotein A-II (apoA-II) ratio were all independently associated with reduction in the risk of incident T2D [38]. Conversely, a recent study of over 5 million nondiabetic adults from the Korean National Health Insurance System cohort reported that a low HDL-C level was associated with a higher risk of developing diabetes over a median follow-up period of 5.1 years, and that this was exacerbated in patients with highly variable HDL-C levels [39]. In another large Korean study of 27,988 subjects with impaired fasting glucose a higher HDL-C was, by contrast, not significantly associated with lower risk of incident T2D over 2.8 years [40]. However, in that study a higher HDL-C/apoA-I ratio was significantly associated with a lower risk of incident T2D [40]. This finding was subsequently validated and extended in a study of Caucasian patients with T2D in whom a higher HDL-C/apoA-I ratio was associated with improved β-cell function and a reduced risk of macrovascular and microangiopathic complications [41]. As HDL particle size is directly proportional to the HDL-C/apoA-I ratio, these results suggest that there is a potential specificity associated with the antidiabetic properties of HDLs, with large particles having superior antidiabetic functions compared to small HDLs. This possibility is clearly worthy of further investigation.

### 2.2. Epidemiological Insights into HDL Subtypes in Diabetes

The HDLs in human plasma comprise several subpopulations of particles that are diverse in terms of size, density, surface charge and composition [42]. Emerging evidence suggests that these HDL subtypes may also be functionally distinct, at least in a cardiovascular disease setting [43,44]. Given that the three most abundant apolipoprotein constituents of HDLs, apoA-I, apoA-II and apoA-IV, all have antidiabetic functions, but that they are not uniformly distributed between all HDL subtypes, it is highly likely that this may also be the case for diabetes [23,25,45,46]. Although it has yet to be investigated systematically, additional evidence that HDL subtypes may be functionally distinct comes from the fact that patients with T1D tend to have HDLs that are larger than those found in healthy people, while small HDLs predominate in people with T2D [47,48,49]. The functional implications of this difference in HDL size has not been explored systematically, but there is some evidence to suggest that the HDLs in T1D and T2D are, indeed, functionally diverse [50].

Type 1 Diabetes

Most patients with T1D have a normal or significantly increased HDL-C level, a reduced total number of HDL particles, and an increased number of large HDL particles relative to that of well-matched, healthy control subjects [48,51]. The functional implications of these differences have been focussed so far on the cholesterol efflux capacity of plasma from these patients, which is consistently enhanced relative to that of nondiabetic controls [48]. While this improvement in HDL function is potentially beneficial, the mechanistic basis of the finding requires further validation because a significant proportion of the increased cholesterol efflux in these subjects is dependent on the ATP binding cassette transporter, ABCA1, which effluxes cholesterol to lipid-free or lipid-poor apoA-I, not to the large HDLs that predominate in these individuals [48]. Collectively, these observations suggest that the large HDL particles in patients with T1D may not be fully functional. It also raises the possibility that HDLs from patients with T1D may have an increased susceptibility to remodelling by plasma factors such as CETP and phospholipid transfer protein, both of which generate lipid-free or lipid-poor apoA-I.

T1D also has a significant impact on the HDL proteome. This area is yet to be explored in detail, but a recent cross-sectional, case–control study of isolated HDLs from young patients with T1D identified significant differences in the protein cargo of HDLs in affected subjects relative to healthy controls [52]. Some of these changes, such as the presence of proteins that are linked to complement activation, appear to be regulated by the extent of glycaemic control, but how this impacts on HDL subpopulation distribution and affects HDL function is not known [52]. It is noteworthy that the cholesterol efflux capacity of the HDLs from the subjects with T1D in that study was comparable to that of healthy controls, an observation that is at odds with the increased efflux in T1D patients that has been reported by Ahmed et al. [48]. The reason for this discrepancy is not clear, but it may reflect differences in average patient age (17 versus 37 years), lifestyle factors (smoking, alcohol intake), and uptake of lipid lowering and increased post translational modifications of HDL apolipoproteins in the older cohort with longer duration diabetes.

Poor glycaemic control in patients with T1D seems to exacerbate changes in HDL subtype distribution. In a small cross-sectional study of 52 adolescents with T1D, those with poor glycaemic control had a lower level of large HDL_2_ particles than those with reasonable glycaemic control, despite having similar HDL-C levels. This difference in HDL subtype distribution suggests that poor glycaemic control reduces the number of HDL particles, but does not impact on HDL function, with cholesterol efflux from hepatoma cells to serum and isolated HDLs being comparable for all of the subjects [53]. In a recent study of T1D patients, by contrast, elevated medium-sized HDL particles and a higher level of HDL-associated paraoxonase (PON) 1 were associated with fewer vascular complications [47]. This result provides further evidence that HDL subclasses in patients with T1D are functionally distinct.

It is important to note that interpretation of all of the above studies is significantly limited by their cross-sectional design. This raises the possibility that the conclusions may be attributable to reverse causality and begs the question of whether any of the observed associations have a causal basis.

Type 2 Diabetes

As ~90% of all patients with diabetes have T2D, there is much more information on the epidemiology of the antidiabetic functions of HDLs in this group than in patients with T1D. The decrease in HDL size in insulin-resistant T2D patients has been reported to correlate with a decrease in the number of large HDL particles and an increase in the number of small HDL particles for a given HDL-C level, indicating that T2D may increase the number of HDL particles [54].

In a large, prospective 13-year study of over 26,000 participants where HDL subclasses were evaluated by NMR spectroscopy in healthy women that subsequently developed type 2 diabetes, small HDLs were positively associated with disease development, while large HDLs were inversely associated with disease development [55]. This result is supported by a more recent, cross-sectional study of over 8000 participants in which the homeostasis model assessment of insulin resistance (HOMA-IR) was found to be inversely associated with HDL_2_ cholesterol levels and positively associated with HDL_3_ cholesterol levels [56]. Similar results were obtained from a smaller, 5-year prospective study in which HDL_2_-C levels were inversely associated with the risk of incident type 2 diabetes, and in another study in which the inclusion of a low plasma apoA-I level improved the power of the established T2D risk prediction models [56,57].

Evidence that insulin resistance may drive HDL subclass distribution towards smaller particles in patients with type 2 diabetes has also been obtained from a small cohort of patients in whom insulin sensitivity was evaluated by hyperinsulinemic clamp, and another prospective study in which HOMA-IR and small HDLs were positively associated with incident diabetes over 7.7-years [54,58]. Whether the relationship between insulin resistance and small HDLs is causal remains to be seen, but it could be postulated that some of the proteins that are selectively transported by small HDL particles inactivate insulin signalling pathways in skeletal muscle and adipose tissue.

A small, prospective study of Japanese Americans, in which a high total HDL-C level was associated with a lower future risk of T2D, further suggested that HDL subtypes may be differentially associated with insulin resistance and T2D risk [59]. Although this association was apparent in patients with a high HDL_2_-C level, but not a high HDL_3_-C level, it was no longer significant after adjusting for visceral adipose tissue area. This suggested that the association may have been mediated by the visceral fat depot, a well-known risk factor for insulin resistance and T2D [60]. However, there is likely to be some validity in this association as an inverse association of a high HDL_2_-C level with T2D risk has also been reported in a much larger, cross-sectional, community-based cohort of 8365 subjects in which a high HDL_2_-C level was associated with reduced insulin resistance, while a high HDL_3_-C level was associated with more severe insulin resistance [56].

The issue of whether or not there is a causal basis for any of these relationships between HDL sand T2D was addressed directly in a Mendelian Randomisation study of over 47,000 participants in the Copenhagen City Heart Study and the Copenhagen General Population Study [61]. The results of that study did not find any evidence of an association of genetic variants with low HDL-C levels and T2D risk [61]. However, that study did not assess the relationship of HDL subtypes, or any aspects of HDL function with T2D risk.

The first direct evidence that the antidiabetic functions of HDLs is causal in humans was obtained from a double-blind, placebo-controlled crossover study of 13 T2D patients, in which plasma HDL levels were transiently increased by the administration of a single intravenous infusion of rHDLs [62]. These patients all sustained a reduction in plasma glucose and an increase in plasma insulin levels, and an overall improvement in glycaemic control [62].

The antidiabetic functions of HDLs are further supported by data from large-scale randomised clinical trials of CETP inhibitors, which chronically increase plasma HDL-C and apoA-I levels. In the Investigation of Lipid Level Management to Understand its Impact in Atherosclerotic Events (ILLUMINATE) trial, treatment with the CETP inhibitor, torcetrapib, improved glycaemic control in statin-treated patients with T2D [12]. A similar result was obtained in the Assessment of Clinical Effects of Cholesteryl Ester Transfer Protein Inhibition with Evacetrapib in Patients with at High Risk for Vascular Outcomes (ACCELERATE) trial, where glycaemic control was found to be improved in T2D patients [63]. Finally, treatment with two other CETP inhibitors, anacetrapib, and dalcetrapib was also found to be associated with a reduced risk of new-onset diabetes in large, randomised clinical outcome trials [14,64]. Insights into the effects of HDL-raising agents on glycaemic control from randomised clinical trials are summarised in Table 1. Whether these beneficial effects are a direct consequence of the increased level of HDLs, or whether they are due to the increased HDL levels counteracting the negative effects of statin treatment in these patients is not known [65].

## 3. Apolipoproteins and the Antidiabetic Functions of High Density Lipoproteins

Early in vitro and preclinical studies have indicated that the three most abundant HDL apolipoproteins, apoA-I, apoA-II and apoA-IV all have antidiabetic properties [23,25]. Conversely, the small, exchangeable apolipoprotein, apoC-III, that is associated with HDLs in normal, healthy people has a potentially adverse effect in patients with diabetes, with lower apoC-III levels being associated with delayed onset of disease [66]. ApoC-III has also been reported to promote β-cell death [67].

### 3.1. Apolipoprotein A-I and Apolipoprotein A-II

The first direct evidence that HDLs and apoA-I have potential therapeutic value in humans with diabetes came from the aforementioned study in which a single infusion of rHDLs prepared with apoA-I and soybean phosphatidylcholine improved glycaemic control in patients with T2D [62]. The basis of the improved glycaemic control in these individuals was attributed to increased secretion of insulin from β-cells and enhanced glucose uptake into skeletal muscle [62]. This result is consistent with what has been reported for apoA-I knockout mice that have impaired glucose tolerance, in mice that overexpress human apoA-I and have improved glucose tolerance, and in in vitro studies of cultured skeletal muscle cells where incubation with lipid-free apoA-I has been reported to increase glucose uptake in an insulin-dependent and -independent manner by increasing glycolysis and mitochondrial respiration [27,28,68,69,70]. Some of these studies are particularly important because they suggest that apoA-I- and HDL-based therapies may improve glycaemic control in patients with T2D that have complete loss of β-cell function and are refractory to many of the currently available antidiabetic therapies [28,71].

Other in vitro studies have revealed that apoA-I and apoA-II in both lipid-free and lipid-associated forms increase insulin synthesis and glucose stimulated insulin secretion (GSIS) in the MIN6 and Ins-1E pancreatic insulinoma β-cell lines [22,23]. The mechanistic basis of these observations involves the activation of a G-protein-cAMP-PKA-FoxO1 pathway, is dependent on the internalization of lipid-free apoA-I into the β-cells, and is associated with the increased expression of the β-cell survival gene, pancreatic and duodenal homeobox 1 (*Pdx1*) [22,72]. The ability of lipid-free apoA-I and apoA-II to increase *Pdx1* gene expression raises the possibility that these apolipoproteins may conserve β-cell function and reduce the adverse effects of activated T-cells in T1D [73,74]. Additionally, all HDL subclasses have been shown to be equally effective at increasing insulin secretion in MIN6 cells [75].

HDLs also protect β-cells from the apoptosis that occurs when blood glucose and free fatty acid levels are increased by endoplasmic reticulum (ER) stress-dependent and -independent mechanisms [76,77]. The ability of HDLs and lipid-free apoA-I to inhibit apoptosis in β-cells has additionally been attributed to reduced expression of the proinflammatory cytokine, interleukin (IL)-1β [78]. While apoA-II is as effective as apoA-I at improving β-cell function in both lipid-free and lipid-associated forms [23], it is not known if it operates through the same mechanisms.

Evidence that the antidiabetic functions of apoA-I and apoA-II translate into improved glycaemic control in vivo is mounting. For example, lipid-free apoA-I treatment increases glucose-stimulated insulin secretion (GSIS) in C57BL6 mice with diet-induced obesity, and in isolated islets from mice with elevated islet cholesterol levels and impaired insulin secretion due to the conditional deletion of the ATP binding cassette transporters, ABCA1 and ABCG1, which export cholesterol from β-cells to lipid-free/lipid-poor apoA-I and HDLs, respectively [24,26,27,79]. However, the precise mechanism by which apoA-I improves β-cell function in this animal model has yet to be elucidated. What we do know is that the observed benefit is not related to the restoration of β-cell cholesterol homeostasis in the case of mice with conditional deletion of ABCA1 and ABCG1 in β-cells [24]. Nor is it related to improved glucose metabolism or to the inhibition of inflammation [24].

ApoA-I also reduces insulin resistance in validated mouse models of T2D. Treatment of insulin-resistant *db/db* mice with lipid-free apoA-I increases glucose uptake by skeletal muscle 1.8-fold [80]. Similar results have also been obtained following lipid-free apoA-I treatment of mice with diet-induced obesity and in rats with pregnancy-induced insulin resistance [26,27,81,82]. The ability of apoA-I to reduce insulin resistance in pregnancy were not, however, confirmed in a recent small, retrospective cross-sectional study of women with gestational diabetes, where serum apoA-I levels were found not to be associated with insulin sensitivity [83]. This discrepancy between the animal and the human studies may be because the apoA-I levels in the animal studies were increased with infusions of unmodified, lipid-free apoA-I, whereas the analyses in the human study were based entirely on differences in endogenous plasma levels of apoA-I that is likely to have undergone post-translational modification and varying amounts of inactivation depending on the duration of the gestational diabetes [50,83]. Whether the infusion of apoA-I that has not been modified in any way and is therefore fully functional into women with gestational diabetes would reduce pregnancy-induced insulin resistance is not known but is a question that is undoubtedly of interest. The evidence that this may be potentially advantageous comes from the increased uptake of glucose by skeletal muscle that has been reported in patients with T2D in whom circulating HDL levels were increased with a single infusion of rHDLs [62]. Whether apoA-II also improves insulin sensitivity by increasing glucose uptake into skeletal muscle is not known. This is a question of considerable interest given that there are few known functions of this highly conserved apolipoprotein.

Lipid free apoA-I treatment has also been shown to increase glucose uptake into the heart in mice [27,81]. These findings have recently been extended in a further mouse study which showed that the ability of apoA-I-containing rHDLs to increase glucose uptake into the heart during myocardial ischemia is associated with reduced cardiac damage [84].

While most investigations into the antidiabetic functions of HDLs and apoA-I have focussed on improving β-cell function and/or insulin sensitivity, there is increasing interest in their effects on α-cells in pancreatic islets. α-cells, the second most abundant cell type in the endocrine pancreas after β-cells, secrete glucagon, which increases blood glucose levels [85]. It has recently been reported that plasma HDL-C levels are inversely associated with fasting glucagon levels in a normal, healthy population [86]. The evidence that this relationship may be causal has been obtained by showing that treating hypoglycaemic mice with isolated HDLs and lipid-free apoA-I reduces glucagon secretion by inhibiting the activation of the PI3K/Akt/FoxO1 signalling pathway in a scavenger receptor B1 (SR-B1)-dependent manner [86].

### 3.2. Apolipoprotein A-IV

ApoA-IV, the third most abundant HDL apolipoprotein after apoA-I and apoA-II, also has antidiabetic functions. This observation was first reported in a landmark study where high-fat fed apoA-IV knockout mice were found to be glucose intolerant, and that glycaemic control in these animals was restored by the administration of recombinant mouse apoA-IV [25]. The benefit of apoA-IV in that study was attributed to improved β-cell function, as evidenced by ex vivo apoA-IV treatment of the isolated islets from apoA-IV knockout mice increasing GSIS [25]. While treatment of apoA-IV knockout mice with apoA-IV did not increase glucose disposal in skeletal muscle in the initial study [25], a more recent study has established that this apolipoprotein does improve insulin sensitivity in wild-type C57BL6 mice by increasing glucose uptake into adipocytes and cardiac muscle [25,87].

### 3.3. Apolipoprotein C-III

ApoC-II is a small, exchangeable apolipoprotein that associates with HDLs in normal, healthy individuals, but is predominantly incorporated into triglyceride-rich lipoproteins in subjects with high plasma triglyceride levels [88]. As a high plasma triglyceride level is a hallmark feature of T2D, but not T1D, it follows that the distribution of apoC-III across plasma lipoproteins will vary according to diabetes type [89].

Epidemiological studies have established that plasma apoC-III levels are positively associated with diabetes, and that *Apoc3/APOC3* gene transcription increases in rat and human hepatocytes under high glucose conditions and is inhibited by insulin [90,91,92,93,94]. Indirect evidence that apoC-III may be causally related to the development of diabetes was obtained from a small randomised, double-blind, placebo-controlled clinical trial of patients with T2D in which treatment with an apoC-III antisense oligonucleotide that reduced plasma apoC-III levels by 88% increased HDL-C levels and improved insulin sensitivity [95]. Treatment with an apoC-III antisense oligonucleotide also reduces circulating apoC-III levels, delays disease onset in a rat model of T1D and improves glucose tolerance in insulin-resistant *ob/ob* mice [66,96].

Mechanistically, the adverse effects of apoC-III in diabetes appear to be related to aberrant Ca^2+^ handling in β-cells, which increases intracellular Ca^2+^ levels [67,96]. Studies in the Ins-1E cell line have indicated that apoC-III causes islet inflammation and β-cell apoptosis by activating mitogen-activated protein kinase (MAPK) and the extracellular signal‑regulated protein kinase ERK1/2 [97]. However, the ability of apoC-III to promote β-cell apoptosis is controversial as it has also been reported to inhibit apoptosis in isolated islets by activating PI3K/Akt signalling, with no effect on MAPK or ERK1/2 [98]. The reasons for these discrepant results are not clear, but may be related to the fact that the isolated islets in the latter study of Storling et al. were preconditioned with apoC-III prior to stimulation with proinflammatory cytokines, which was not the case in the other investigations [67,96,97]. It is also possible that these discrepant results reflect fundamental differences in the processes that are mediated by apoC-III in Ins-1E cells and primary islets.

Irrespective of whether apoC-III is pro- or antiapoptotic, the currently available insights indicate that this apolipoprotein has the potential to impact adversely on glycaemic control in patients with T1D and T2D by reducing β-cell function. Whether apoC-III also adversely impacts on insulin sensitivity has not been investigated directly. This is clearly an area that warrants further investigation at the preclinical stage as well as in human studies.

## 4. Diabetes and the Regulation of High Density Lipoprotein Function

The lipid and apolipoprotein constituents of HDLs are both susceptible to modifications that have the potential to impact adversely on the antidiabetic and cardioprotective functions of HDLs. Although this is an area of considerable importance, it has not been investigated systematically, with many key questions remaining unanswered. As a result, little progress has been made in recent years in identifying therapeutic targets that may inhibit these modifications in patients with diabetes. This is clearly an area worthy of investigation going forward.

### 4.1. Nonenzymatic Glycation

The nonenzymatic glycation of apoA-I that occurs as a consequence of spontaneous interaction with reactive α-oxoaldehydes generates a diverse array of advanced glycation end-products (AGEs) including Nε-(carboxyethyl) lysine, Nε-(carboxymethyl) lysine and Nω-(carboxymethyl) arginine and is an extensively studied HDL modifications in diabetes [99,100,101]. AGE formation is particularly prevalent in patients with poor glycaemic control [50].

Nonenzymatic glycation of apoA-I impairs several of the cardioprotective functions of HDLs, including their ability to accept the excess cholesterol that effluxes from macrophages in the artery wall, the process that drives the first step in the reverse cholesterol transport pathway [71,102,103,104,105,106]. However, impaired cholesterol efflux has not been reported in all patients with diabetes. For example, in a cross-sectional study of 552 subjects that included 288 controls, 126 subjects with impaired glucose tolerance and 138 subjects with T2D, cholesterol efflux was comparable across all three groups of subjects [107]. While the cross-sectional design of that study and the potential for reverse causality may explain this discrepant result, it also raises the possibility that there may be a threshold level of nonenzymatic glycation of apoA-I and other apolipoproteins, below which the HDL function is not compromised.

**Figure 1:**
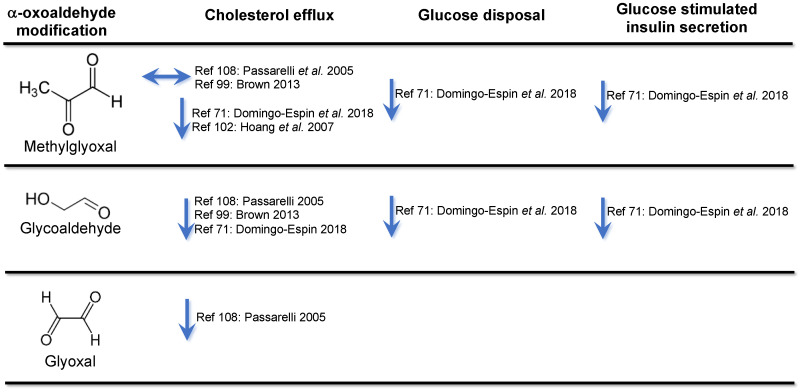
Nonenzymatic glycation of apoA-I impairs HDL function.

It should, however, be noted that only selected reactive α-oxoaldehyde-mediated modifications to apoA-I reduce cholesterol efflux. For example, methylglyoxal, one of the most abundant α-oxoaldehydes in diabetic plasma nonenzymatically glycates apoA-I extensively but does not affect the capacity of apoA-I to efflux cholesterol from fibroblasts or macrophages (Figure 1) [108]. Glycoaldehyde and glyoxal, by contrast, both nonenzymatically glycate apoA-I and have been reported to markedly impair its ability to promote cholesterol efflux from macrophages by destabilising ABCA1 (Figure 1) [108]. The result for glycoaldehyde was, by contrast, not confirmed in a more recent study using macrophages [99]. The reason for this discrepant result is not clear but may be related to the different glycoaldehyde concentrations that were used in the studies. CYP7A1 and RAGE also reduce the ability of ABCA1 to efflux cholesterol from macrophages to apoA-I in patients with T2D [109,110]. Nonenzymatic glycation additionally prevents apoA-I from inhibiting one of the initiating events in atherosclerotic lesion development: the recruitment of human monocytes to the endothelial surface [102]. rHDLs that contain nonenzymatically glycated apoA-I also have a reduced capacity to increase glucose uptake into cultured skeletal muscle cells and improve insulin secretion from Ins-1E cells [71].

ApoA-I that has been nonenzymatically glycated glycated by the reactive α-oxoaldehydes, glycoaldehyde and glyoxal, has an impaired ability accept cholesterol from cells. Nonenzymatic glycation of apoA-I with the reactive α-oxoaldehyde, methylglyoxal, has variable effects on cholesterol efflux. Nonenzymatic glycation impairs the ability of apoA-I to mediate glucose disposal in skeletal muscle and increase insulin secretion in response to glucose in β-cells.

While mechanistic insights into the reduced efflux of cholesterol from mouse J774 macrophages to HDLs under conditions that favour nonenzymatic glycation have focussed on apoA-I [71], extensive post-translational modification to other components of the HDL proteome, including deamidation and carbonylation and impaired lipid binding capacity may also contribute significantly to the loss of HDL function in diabetes [71,99,111,112].

Importantly, the adverse effects of nonenzymatic glycation on some of the cardioprotective functions of apoA-I, including the efflux of cholesterol from THP-1 macrophages, can be reversed by inhibiting glycation with aminoguanidine, and by reducing AGE levels with the cross-link breaker alagebrium chloride [102]. Alagebrium as well as the insulin sensitiser metformin, and pyridoxamine which inhibits AGE formation and scavenges reactive oxygen species (ROS) also conserve the cardioprotective functions of apoA-I, including its ability to activate lecithin:cholesterol acyltransferase (LCAT), the enzyme that acts on HDLs and generates almost all of the cholesteryl esters in plasma [101]. However, these agents cannot reverse the nonenzymatic glycation of apoA-I, or restore LCAT activity once it has been compromised [101]. Nonenzymatic glycation of lipid-free and lipid-associated apoA-I also inhibits the anti-inflammatory properties of rHDLs and HDLs from patients with diabetes by reducing the ability of the particles to inhibit ROS formation in endothelial cells [50,113].

### 4.2. Oxidative/ER Stress

Oxidative stress leading to the formation of ROS is a hallmark feature of diabetes [114]. The activity of the HDL-associated antioxidant enzyme, PON1, is decreased in HDLs that contain nonenzymatically glycated apolipoproteins, making the particles less able to counteract the pro-oxidant environment that characterises the diabetic state [115,116,117]. Phospholipids and triglycerides are also more readily hydrolysed in HDLs that contain nonenzymatically glycated apolipoproteins, leading to increased free fatty acid levels and oxidation that increase the susceptibility of β-cells to failure [115,116,117,118].

HDLs that contain nonenzymatically glycated apolipoproteins also have a reduced capacity to inhibit the oxidation of low density lipoproteins (LDLs) [115,116,119]. As the accumulation of oxidised LDLs in the artery wall is a key event in atherosclerotic lesion development, it follows that nonenzymatically glycated apoA-I may contribute indirectly to the accelerated atherosclerotic lesion development that occurs in diabetes [120]. Conversely, as oxidised LDLs inhibit insulin gene transcription and promote β-cell apoptosis in isolated human and rat islets, and in MIN6 and Ins-1E cells, it follows that the capacity of unmodified HDLs (i.e., those that do not contain nonenzymatically glycated apolipoproteins) to conserve β-cell function and insulin content is also related to inhibition of LDL oxidation [121].

Nonenzymatically glycated HDLs also have reduced sphingosine-1-phosphate (S1P) levels, a modification that has been implicated directly in accelerated death of rat cardiomyocytes [122]. S1P is a bioactive lipid that, in association with apolipoprotein M, protects endothelial cells from apoptosis, inflammation and oxidative stress, and reduces the tissue damage that occurs in the heart following ischemia/reperfusion injury [78,123,124]. The reduction of HDL S1P levels in subjects with diabetes has also been implicated as a driver of impaired endothelial function, and is thus a further cause of the accelerated atherosclerotic lesion development that prevails in patients with diabetes [125].

### 4.3. Inflammation

The ability of HDLs to inhibit inflammation in macrophages and endothelial cells, one of the hallmark cardioprotective functions of these lipoproteins, is compromised in patients with diabetes. The inability of HDLs isolated from patients with diabetes to reduce cytokine-activated adhesion molecule expression in endothelial cells has been observed in large HDLs from patients with T1D even though these particles have elevated S1P and apoM levels [126]. This somewhat contradictory result has been attributed to a change in the conformation of apoM that results in an inability of the S1P/apoM complex on large HDL particles to activate the PI3K/Akt pathway [126]. The selective nonenzymatic glycation of lysine residues in apoA-I also reduces the ability of the apolipoprotein to inhibit inflammation in THP-1 macrophages [127]. The mechanistic basis of this observation has been attributed to an alteration in the conformation of the nonenzymatically glycated apoA-I that reduces its binding to the macrophage surface [127].

It is noteworthy that the impaired anti-inflammatory effects of HDLs in patients with diabetes can be restored with a single infusion of unmodified rHDLs that have not been exposed to a proinflammatory, pro-oxidant environment [128]. In a small study of patients with T2D that received a single rHDL infusion that transiently increased circulating HDL levels, the ability of the plasma HDL fraction to inhibit inflammation in the endothelial cells and the binding of monocytes to fibrinogen both improved, as did the ability of the patient plasma to efflux cholesterol from macrophages [128].

## 5. Conclusions

A plethora of preclinical and mechanistic evidence indicating that HDLs have antidiabetic functions and improve glycaemic control by increasing insulin sensitivity and β-cell function has emerged in the last decade. The clinical relevance of these studies has been consolidated by the outcomes of several randomised clinical trials where increasing HDL-C levels in T2D patients with a CETP inhibitor or with an rHDL infusion is associated with improved glycaemic control. While these results do not inform on causality, the consistency of this association is generating significant interest in the development of new therapies for increasing HDL levels in patients with T2D. The compelling “proof-of-principal” evidence in support of interventions that increase HDL levels as being beneficial for patients with T2D that are refractory to currently available antidiabetic agents is compelling. Although it is not known whether raising HDL levels in patients with T1D would also be beneficial, this is an area that is definitely worthy of investigation. The challenge going forward will be how to take advantage of these findings by devising innovative approaches for developing antidiabetic agents that increase HDL levels or that mimic the antidiabetic properties of HDLs and their main apolipoproteins.

## Figures and Tables

**Table 1:** Summary of randomised clinical trials demonstrating the antidiabetic functions of HDLs in humans.

Intervention	n	Impact on HDL-C	Impact on T2D	Reference
***Reconstituted HDL Infusion***				
rHDL	13	↑33 ± 4.3%	Reduced plasma glucose Increased plasma insulin	Ref [62]: Drew et al., 2009
***CETP inhibition***				
Evacetrapib (ACCELERATE)	8236	↑131.9 ± 56%	Decreased HbA1c	Ref [63]: Menon et al., 2020
Anacetrapib (REVEAL)	30,449	↑152.8 ± 1.6%	Reduced risk of new-onset diabetes	Ref [14]: HPS TIMI REVEAL Collaborative Group
Torcetrapib (ILLUMINATE)	15,067	↑72.1 ± 34.7%	Decreased glucose Decreased insulin Decreased insulin resistance Decreased HbA1c	Ref [12]: Barter et al., 2007
Dalcetrapib (dal-OUTCOMES)	15,871	↑33.9 ± 2.8%	Reduced risk of new-onset diabetes in acute coronary syndrome patients	Ref [13]: Schwartz et al., 2020
